# An insight into the sialotranscriptome and virome of Amazonian anophelines

**DOI:** 10.1186/s12864-019-5545-0

**Published:** 2019-03-04

**Authors:** Vera Margarete Scarpassa, Humbeto Julio Debat, Ronildo Baiatone Alencar, José Ferreira Saraiva, Eric Calvo, Bruno Arcà, José M. C. Ribeiro

**Affiliations:** 1 Laboratório de Genética de Populações e Evolução de Mosquitos Vetores de Malária e Dengue, Coordenação de Biodiversidade, Instituto Nacional de Pesquisas da Amazônia, Manaus, Amazonas Brazil; 2 Instituto de Patología Vegetal, Centro de Investigaciones Agropecuarias, Instituto Nacional de Tecnología Agropecuaria (IPAVE-CIAP-INTA), Córdoba, Argentina; 3 Department of Public Health and Infectious Diseases, Division of Parasitology, Sapienza University of Rome, Rome, Italy; 4 Laboratory of Malaria and Vector Research, National Institute of Allergy and Infectious Diseases, Bethesda, MD USA

**Keywords:** Vector biology, Mosquitoes, Malaria, Virus, Salivary glands, Transcriptome

## Abstract

**Background:**

Saliva of mosquitoes contains anti-platelet, anti-clotting, vasodilatory, anti-complement and anti-inflammatory substances that help the blood feeding process. The salivary polypeptides are at a fast pace of evolution possibly due to their relative lack of structural constraint and possibly also by positive selection on their genes leading to evasion of host immune pressure.

**Results:**

In this study, we used deep mRNA sequence to uncover for the first time the sialomes of four Amazonian anophelines species (*Anopheles braziliensis, A. marajorara, A. nuneztovari* and *A. triannulatus*) and extend the knowledge of the *A. darlingi* sialome. Two libraries were generated from *A. darlingi* mosquitoes, sampled from two localities separated ~ 1100 km apart. A total of 60,016 sequences were submitted to GenBank, which will help discovery of novel pharmacologically active polypeptides and the design of specific immunological markers of mosquito exposure. Additionally, in these analyses we identified and characterized novel phasmaviruses and anpheviruses associated to the sialomes of *A. triannulatus*, *A. marajorara* and *A. darlingi* species.

**Conclusions:**

Besides their pharmacological properties, which may be exploited for the development of new drugs (e.g. anti-thrombotics), salivary proteins of blood feeding arthropods may be turned into tools to prevent and/or better control vector borne diseases; for example, through the development of vaccines or biomarkers to evaluate human exposure to vector bites. The sialotranscriptome study reported here provided novel data on four New World anopheline species and allowed to extend our knowledge on the salivary repertoire of *A. darlingi*. Additionally, we discovered novel viruses following analysis of the transcriptomes, a procedure that should become standard within future RNAseq studies.

**Electronic supplementary material:**

The online version of this article (10.1186/s12864-019-5545-0) contains supplementary material, which is available to authorized users.

## Background

Anopheline mosquitoes (Diptera: Culicidae: Anophelinae) of the *Anopheles* Meigen, 1818 genus are important in public health because they are vectors of human malaria parasites in addition to arboviruses. In Brazil they are popularly known as “muriçoca”, “mosquito prego”, “suvela”, “pernilongo”, and “carapanã” [1]. Their development comprise the stages of egg, larvae (four instars), and pupae, which are aquatic, while the adult stage is terrestrial. Both male and female adults feed on carbohydrates from flowers and fruits; however, only females are hematophagous, using the proteins found in host blood for the production and development of their eggs [2]. While feeding blood, they can transmit pathogens to their hosts.

Currently, the *Anopheles* genus includes 465 formally recognized species, which are subdivided into seven subgenera: *Anopheles* (cosmopolitan, 182 species), *Baimaia* (Oriental, one species), *Cellia* (Old World, 220 species), *Kerteszia* (Neotropical, 12 species), *Lophopodomyia* (Neotropical, six species), *Nyssorhynchus* (Neotropical, 39 species), and *Stethomyia* (Neotropical, five species) [3]. Worldwide, the primary vectors of human malaria parasites belong to the subgenera *Anopheles*, *Cellia*, *Kerteszia* and *Nyssorhynchus.*

In the Americas, the dominant vector species belong to the *Anopheles* (three species) and *Nyssorhynchus* (six species) subgenera [4]. Among species of the subgenus *Nyssorhynchus*, *Anopheles darlingi* is the primary vector in Brazil, particularly in the Brazilian Amazon, and in other countries in South America [4, 5]. The remaining dominant vector species are *A. albimanus*, a member of the *A. albitarsis* complex, *A. marajoara*, *A. aquasalis,* and *A. nuneztovari* [4]. Other species of the *Nyssorhynchus* subgenus may be secondary local vectors or were found naturally infected with malaria parasite, such as *A. benarrochi*, *A. rangeli*, *A. oswaldoi* s.l., *A. strodei*, *A. rondoni*, *A. trinkae*, *A. braziliensis*, *A. triannulatus,* and *A. mattogrossensis* [6, 7].

*Anopheles darlingi* is one of the most anthropophilic and efficient malaria vector in the Neotropical region, particularly in the Brazilian Amazon region [5, 8]. It is mainly a riverine mosquito, amply distributed in the rainforest but also it is found in other regions from Brazil, with exception of the dry areas of northeastern region. *Anopheles darlingi* also efficiently adapts in areas of deforestation and altered environments, favoring its abundance and expansion and consequently triggering malaria outbreaks. Adults of this species bite throughout the night [7, 8], however, often two biting peaks have been observed, one at sunset and the other at dawn. Specimens of *A. darlingi* have been captured in both indoor and outdoor environments, with predominance for the later [8].

*Anopheles marajoara* is a member of the *A. albitarsis* complex. In the past, it was believed that *A. marajoara* was a secondary or local vector of minor importance. However, studies conducted in peri-urban areas of the city of Macapá, in the state of Amapá, Brazil, demonstrated that it can be a significant regional vector [9–11] as well as in Boa Vista, in the state of Roraima [12]. Supporting these findings, in the District of Coração, state of Amapá, *A. marajoara* was the most frequent species, together with *A. darlingi* and *A. braziliensis*, showing anthropophilic behavior and being captured in both indoor and outdoor environments [13, 14].

Previous studies have reported *A. braziliensis* as a zoophilic species with little or no importance in malaria transmission [1, 15] or as a secondary vector [5]. It has, however, been found infected with human malaria parasites in the states of Amazonas [8, 16], Amapá [9], Rondônia [17], and Roraima [12]. Curiously, in the District of Coração, state of Amapá, *A. braziliensis* was one of the three most abundant and anthropophilic species. It was captured in both indoor and outdoor environments, although it was more abundant outdoors [14]. Thus, *A. braziliensis* may play some role in malaria transmission when at high density.

*Anopheles triannulatus* sensu lato is predominantly zoophagic, exophilic, and is often found in the forest of the Brazilian Amazon region, but also it is easily found in the edge of forests. However, *A. triannulatus* s.l. has been reported with human malaria parasites in different regions of Brazil [7, 11, 18], as well as Peru and Venezuela [19, 20]; however, due to its behavior and habitat it has been recognized as a secondary vector.

Currently, *Anopheles nuneztovari* is recognized as a species complex, where *A. nuneztovari* s.s. is incriminated as an important human malaria vector in Colombia and Venezuela, showing endo and exophagic behaviors, and elevated levels of anthropophily and infection rate [21, 22]. On the other hand, the Brazilian populations of *A. nuneztovari* s.l. are predominantly zoophagic; however, this species has been reported to be infected with *Plasmodium* spp. in five states of the Brazilian Amazon region [6, 7, 23], and it was incriminated as a local vector in the state of Amapá, Brazil [11]. Based on recent studies, *A. nuneztovari* s.l. may consist of two or more species within the Brazilian Amazon [24, 25], likely with different susceptibility to malaria parasites.

From the five species mentioned above, only *A. darlingi* has been studied under genomic and proteomic approaches, including transcriptome salivary gland analyses [26, 27]. In this study, we present next generation sequencing and sialotranscriptome approaches to investigate the salivary protein composition of five anopheline species from the Brazilian Amazon region, which include two samples of *A. darlingi*, an important malaria vector in this region, *Anopheles marajoara*, an important local malaria vector, and *Anopheles nuneztovari*, *Anopheles triannulatus,* and *Anopheles braziliensis*, secondary malaria vectors. Additionally, we discuss the evolutionary aspects of these secreted salivary compounds with other anopheline salivary protein previously studied. These protein sequences may help identification by mass spectrometry of polypeptides of pharmacological interest, or to help in the design of immunological markers of vector exposure.

The *Anopheles* virus landscape has been scarcely studied. The O’nyong-nyong arbovirus is the only *Anopheles* vectored virus yet reported, and the insect specific viruses of most Anophelines have been barely explored [28]. Here, by assessing mosquito sialotranscriptome data, we were able to identify and characterize three novel viruses associated to *A. triannulatus*, *A. marajoara,* and *A. darlingi* species. The detected viruses correspond to emergent clades of insect-specific viruses, predominantly hosted by mosquitoes.

## Results and discussion

### General aspects of the assemblies

**Fig. 1:**
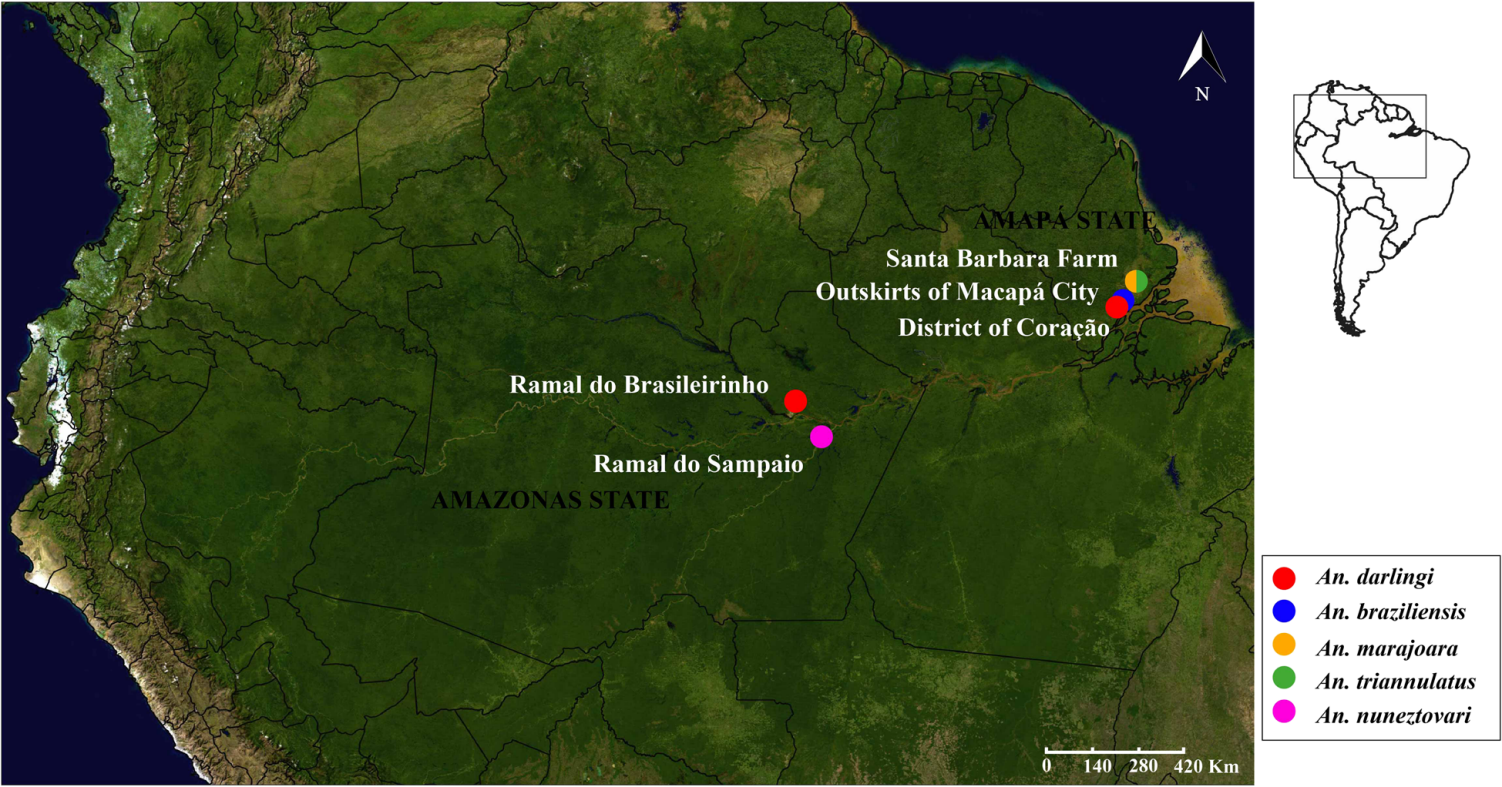
Map showing the locations sampled for anopheline mosquitoes. The map was obtained from the site SimpeMappr, http://www.simplemappr.net/#tabs=6 and is on public domain

The five anopheline species used in this study were collected in the Brazilian Amazon region (see Fig. 1 and Additional file 1: Table S1). Following extraction of salivary RNA of the organisms using RNeasy, and determination of their quality by a Bioanalyzer Nano Chip, messenger mRNA was purified using an oligo-dT protocol. This was used to make the cDNA libraries using the NEBNExt Ultra Directional RNA kit, which were sequenced in an Illumina Hiseq 2500 DNA sequencer (for details see the methods section). Over 115 million reads were obtained for each of the *A. darlingi* libraries, and from 41 to 76 million reads for the other 4 libraries (Additional file 1: Table S2 and S3). From ~ 6 to 38 thousand CDS were extracted from each of the five assemblies, varying in average from 615 to 1200 nt in length (Additional file 1: Table S4).

### Classification of transcripts coding for putative secreted proteins

#### General considerations

Based on our previous review on the sialome of nematocera blood suckers [29], as well as on the most recent analysis of anopheline sialomes [30], we classified and retrieved 723 full length or near full length transcripts from the five anopheline species under study. After removing some redundant sequences (by restricting sequences within 98% identity), these were reduced to 593 sequences, partitioned among the species as follows: *A. braziliensis*, 126; *A. darlingi,* 102; *A. marajoara,* 170*; A. nuneztovari,* 106; *A. triannulatus,* 89. These sequences are available in the hyperlinked Additional file 2: Spreadsheet S1. Additional file 1: Table S5 summarizes these sequences classified into their families, average expression indexes and references to their functional status, when known.

From inspection of Additional file 1: Table S5, it is evident that the most expressed transcripts code for gVAG proteins, which are members of the antigen-5 family (of unknown function) [31, 32], and the 30 kDa antigen/Aegyptin protein family, an inhibitor of collagen-induced platelet aggregation [33–36]. The D7 family (acting as kratagonists of biogenic amines and lipid mediators of hemostasis) [37–40], and the anti-thrombin peptide family (cE5/anophelin) are also well expressed [41–45], as are the ATP/ADP hydrolyzing enzymes apyrase/5’nucleotidase (inhibitors of ADP-induced platelet aggregation) [46, 47] and peroxidases, shown to inhibit catecholamine vasoconstrictor effects [48, 49].

#### Apyrases/5′-nucleotidases

When the EI for members of the apyrase/5′-nucleotidase-coding transcripts are analyzed (Additional file 1: Table S6), two groups of transcripts are apparent, one with EI larger than 25 and another with values smaller than 11. Alignment of their protein sequences with other anopheline proteins from [30] (Additional file 3: Figure S1) shows two distinct clades, named 5Nuc and Apy in the figure, with New World (NW) and Old World (OW) as sub clades. Notably, all products associated with high EI values clustered in the 5Nuc clade, while those with smaller values clustered in the Apy clade (Additional file 3: Figure S1).

The salivary apyrase of *Aedes aegypti*, belonging to the 5′-nucleotidase superfamily, is the only mosquito salivary apyrase thus far characterized biochemically. The coding gene product was found following chromatographic purification of the activity followed by tryptic digestion of the enzyme, Edman degradation of the products, and PCR-based transcript identification [47]. The product was later cloned and recombinantly expressed, with verification of the apyrase and platelet inhibitor activities [50]. In anophelines the apyrase gene was apparently duplicated early in the anopheline evolution to produce 2 genes, named apyrase and 5′-nucleotidase [31, 32, 51, 52]. However, the substrate specificities of these two enzymes are so far unknown. Perhaps the two enzymes both work as a typical apyrase, hydrolyzing ATP to AMP, or perhaps only one has apyrase activity while the other further hydrolyzes AMP to adenosine. These experiments remain to be done.

### Peroxidases

While Old World anophelines appear to have one gene coding for a salivary peroxidase, New World species appear to have multiple copies of salivary peroxidase-encoding genes [30]. Indeed, the phylogeny reconstruction of the known anopheline peroxidase sequences including those disclosed in this work shows multiple peroxidases for all New World anophelines, including 2 distinct clades for *A. darlingi* containing 6 sequences deriving from at least 3 genes (Additional file 3: Figure S2). Interestingly, two sequences, one from *A. albimanus* and the other from *A. braziliensis,* form a robust subclade within a clade from solely Old-World species, named NW in Additional file 3: Figure S2. These clades also grouped sequences that have similar expression indexes, as indicated by the symbols +, ++ or +++ assigned from low, medium, or high expression indexes.

### D7 family

This family contains one of the first cloned salivary protein from a mosquito [53] and was later found to belong to the odorant binding superfamily [54]. D7 proteins function as kratagonists (agonist binders) of biogenic amines and eicosanoid mediators of inflammation [37]. One *A. stephensi* protein named hamadarin was shown to inhibit blood clotting [55]. Several of these proteins have been crystalized [38–40]. In *A. gambiae* there are 3 genes coding for 2 domain proteins (large D7 proteins), and 5 genes coding for short D7 proteins (single domains) [56], arranged in tandem. Interestingly, the last gene coding for the long or short protein was poorly expressed, and it was proposed they may be turning into pseudogenes. The alignment of the long D7 proteins of the Amazonian anophelines with the known anopheline homologues shows a clear clade of New World genes coding for D7-L1 and D7-L2, but no genes coding for the D7-L3 proteins (Additional file 3: Figure S3). Note that the D7-L3 of the New World species *A. darlingi* and *A. albimanus,* shown in the figure, were previously deducted from their genomes. Previous analysis of the *D7* gene cluster in anophelines also, suggested that *D7L1* was lost in *A. darlingi* and *A. albimanus,* as well as in other Old World anopheline species [30], It is likely this is the case for the four new Amazonian species analyzed here, or, more presumably, the gene was lost by their common ancestor. In fact, the two *A. marajoara* and *A. braziliensis* sequences shown in the phylogenetic tree (Additional file 3: Figure S3) and the additional ones reported in the hyperlinked spreadsheet S1 may be allelic variants.

The alignment of the short D7 proteins, named D7r1 through D7r5, also shows the inexistence of gene expression of the D7r5 from the Amazonian anophelines as deducted from their transcriptomes, although they were found in the genomes of *A. darlingi* and *A. albimanus* [30]. Two related transcripts from *A. nuneztovari* were found expressed with EI between 1 and 2, although their counterparts are better expressed, with EI values above 10% (Additional file 3: Figure S4).

### Antigen 5 family

The antigen 5 family is ubiquitously found in sialotranscriptomes of blood sucking arthropods [57]. Several members of this family have been described in anophelines and other mosquitoes, but no mosquito protein of this family has been functionally characterized. In *Stomoxys calcitrans,* a salivary antigen 5 protein has been found to bind to the Fab region of immunoglobulins and could inhibit complement activation [58, 59]. A recombinant protein from the triatomine bug, *Dipetalogaster maxima,* displays superoxide dismutase activity and inhibits platelet activation by low doses of collagen [60]. An antigen 5 protein expressed in the salivary glands of the horse fly, *Tabanus yao,* exceptionally acquired a disintegrins RGD domain, as well as a hydrophobic pocket that efficiently scavenges leukotrienes; thus defining a bifunctional activity of platelet aggregation inhibitor and leukotriene kratagonist [61, 62]. With this diverse list of functions, it is difficult to predict the adaptive value of these proteins in mosquitoes.

The alignment of the deducted Amazonian anopheline proteins belonging to the antigen 5 family, together with those previously described for anopheline mosquitoes [30], shows that all transcripts cluster within the gVAG clade, while none have been found in the other six clades (Additional file 3: Figure S5). Notice also that the transcripts coding for these proteins are highly expressed, many having EI values above 50%, reaching 100% in *A. darlingi* and *A. braziliensis*, meaning it is the most abundant transcript found in the sialotranscriptome of these species.

### 30 kDa antigen/aegyptin family

The 30 kDa antigen from *Ae. aegypti* was the first described member of this family [63], and it was later characterized as an inhibitor of collagen-induced platelet aggregation [33, 34]. Anopheline members of this family were identified as glycine-acidic rich proteins [56], later shown to be related to aegyptin, and also to inhibit collagen-induced platelet aggregation [64]. Proteins of this family have a more complex amino terminus and a less complex glycine rich, acidic, carboxy terminus. A protein named Simplagrin was found in salivary transcriptomes of black flies; very divergent from mosquito aegyptins, but conserving the general structure of a complex followed by a less complex amino acid sequence and was also able to inhibit collagen-induced platelet aggregation [65], indicating this protein family was present _~_ 150 million years ago in the blood-feeding precursors of black flies and mosquitoes. This protein family is unique to Culicoidea. While *Aedes* mosquitoes have more than one gene of this family expressed in their salivary glands [66, 67], anopheline mosquitoes appear to have only one [30, 56, 68–70]. The sialotranscriptome of Amazonian anophelines indicate these mosquitoes also have a single gene, with possible exception of *A. nuneztovari*, all with high expression indices (Additional file 3: Figure S6). Whether the two genes from *A. nuneztovari* result from a gene duplication, or whether it derives from single gene from two cryptic subspecies, remain to be determined.

### SG7/Anophensin/Albicin family

The uniquely anopheline family named SG7 was first discovered in a salivary transcriptome of *A. gambiae* [31, 32]. A second member of this family was later discovered in *A. gambiae* [56]. Other anopheline species were also shown to express this protein family [26, 69, 70]. A member of the family from *A. stephensi* was characterized as an inhibitor of the kallikrein/bradykinin pathway and named anophensin [71]. Interestingly one SG7 member from *A. albimanus* had demonstrated anti-complement activity and was named albicin [72]. More recently, salivary gland homogenates of *A. aquasalis* were shown to have a similar activity [73]. Interestingly, this family appears to have originated from a gene duplication from an aegyptin-coding gene that evolved to the present day SG7 [30, 74]. Additional file 3: Figure S7 depicts the evolutionary relationships between the known members of the SG7 family with those disclosed in this study.

### Sg1 family

The SG1 family, exclusive of anophelines, is related to the 62/34 kDa family of culicines, most arranged as single exon genes and postulated to be acquired by horizontal transmission from a bacterial host early in the evolution of Culicidae [29]. The function of any member of this family is still unknown. Seven members are recognizable in *A. gambiae*, but some (SG1 and SG1a) were missing in *A. darlingi* and *A. albimanus* [30]. The phylogram depicted in Additional file 3: Figure S8 shows the lack of SG1 and SG1a members in all New World species. The figure also shows many variants of the SG1 sub-families within the New World species, but this may be due to allelic variation in the same gene rather than reflecting gene duplications.

### cE5/anophelin family

This is a uniquely anopheline family coding for acidic polypeptides, containing a signal peptide indicative of secretion and a mature molecular weight of ~ 6.5 kDa. The cE5 polypeptide was described during the first sialotranscriptome of *A. gambiae* [32], while the polypeptide named anophelin was described as the *A. albimanus* thrombin inhibitor [41, 42]. Alignment of the known members [30] of this family with those found in the present study produces the phylogram shown in Additional file 3: Figure S9. The Amazonian anopheline transcripts coding for members of this family are reasonably well expressed, with EI values ranging from 8 to 57.

### Basic tail family

This family of secreted polypeptides was first described in *Aedes* where it has a doublet of positively charged amino acids in its carboxy terminus. However, in *A. darlingi* it was found a homolog without the basic doublet, and similar proteins were found in other Old World anopheline species [29], Additional file 3: Figure S10 shows the alignment of culicine and anopheline sequences, the deducted amino acid motif and the phylogenetic tree derived from the alignment. Despite being a reasonably simple motif, when used to search matches of the non-redundant protein database from the NCBI, with over 160 million entries, only mosquito proteins were retrieved.

### Synonymous and non-synonymous single nucleotide polymorphisms

After mapping the reads to the assembled transcripts of each species transcriptome, it becomes possible to evaluate the rate of synonymous and non-synonymous polymorphism among the different protein classes. Previously we observed an increased rate of non-synonymous polymorphisms in salivary secreted proteins when compared to salivary housekeeping proteins, using transcriptomes from ticks and hematophagous insects [75–78]. Following selection of transcripts that have 100 or more read depth coverage, and with more than 15 transcripts in each functional class, it is observed that the secreted and the unknown class have the highest rate of non-synonymous to synonymous polymorphism (Additional file 1: Table S7). This is not an artifact of excess coverage of the transcripts of the secreted class, as the protein synthesis class has high coverage depth and has one of the lowest rates of non-synonymous to synonymous mutations. The high non-synonymous rate observed for the secreted class may reflect a reduced mutational constraint of these proteins, and additionally they may be under positive selection to escape their hosts’ immune pressure [79].

### Novel Amazonian anophelines viruses

Metatranscriptomic surveys of diverse invertebrate taxa have been successfully employed to reveal an astonishing diversity of RNA viruses [80]. Here, in parallel to the characterization of the endogenous profile of Anophelines sialotranscriptomes, we effectively identified adventitious RNA sequences corresponding to three novel viruses associated to *A. triannulatus*, *A. marajoara,* and *A. darlingi*. The detected viruses correspond to emergent clades of insect-specific negative-strand single RNA viruses, predominantly hosted by mosquitoes: *Orthophasmavirus* (*Bunyavirales*) and *Anphevirus* (*Mononegavirales*).

### *Anopheles triannulatus* orthophasmavirus

The *Orthophasmavirus* genus (*Phasmaviridae*) was established by the discovery of two viruses in phantom midges (family *Chaoboridae*) from North America: Kigluaik phantom orthophasmavirus (KPOPV) and Nome phantom orthophasmavirus (NOMV) [81]. In turn, four additional viruses which clustered with KPOPV and NOMV were identified in mosquitoes and cockroaches from China [82] and dubbed Wuhan mosquito virus 1 and 2 (WMV1 & WMV2), Shuangao insect virus 2, and Wuchang cockroach virus 1, and incorporated to the genus. Furthermore, three similar viruses derived from metagenomic libraries of magpie moths and pooled Odonata species appear to be related to phasmaviruses [80]. Evolutionary studies suggest that phasmaviruses are sister clades of joniviruses and feraviruses with similar genomic architecture. Phasmaviruses are multipartite ssRNA(−) viruses presenting three genome segments: S, M, and L ranging from 1.8–2.2, 2.0–2.8, and 6.5–6.7 kb in length [83]. Phasmaviruses encode nucleoproteins (S segment), glycoproteins (M segment), and RNA-dependent RNA polymerase (RdRPs) (L segment). Additionally, the S segments appear to encode also NSs proteins at equilocal position with joniviruses. In order to investigate the eventual presence of viral RNA in our datasets, we subjected de novo assemblies to bulk BLASTX searches against the NCBI refseq viral protein database. Interestingly, a 6186 nt contig from *A. triannulatus* showed a significant hit (56% amino acid (aa) identity; E-value = 0, query coverage = 99%) with the RdRP of WMV1. Further read mapping and polishing extended the contig into a 6494 nt sequence (RNA 1), supported by 69,379 reads (mean coverage = 1328X), encoding a single 6339 nt ORF. A 2112 aa protein was predicted, presenting a Bunyavirus_RdRP functional domain (910–1225 aa coordinates; pfam04196, E-value = 4.81e-06) (Additional file 3: Figure S11A). To explore this putative RdRP, multiple alignments were generated with representative polymerases of *Bunyavirales* type species (Additional file 3: Figure S11.B). All major catalytic motifs A-C, including the SDD motif of segment-negative stranded RNA viruses, pre motif A, motif D-E, and conserved N-terminal domain region were detected, suggesting that the predicted RdRP could be functional. In addition, it became evident that the predicted replicase shared significant similarity with the RdRP of the *Orthophasmavirus* KPOPV. In this scenario, KPOPV segments M and S were queried against the *A. triannulatus* sialotranscriptome, and two contigs of 2061 nt and 996 nt were retrieved which were curated into a 2246 nt (RNA 2, mean coverage = 396X) and 2069 nt (RNA 3, mean coverage = 174X). RNA 2 presents a single 2052 nt ORF encoding a putative glycoprotein similar to that of WMV1 (51% aa identity; E-value = 0, query coverage = 99%). The putative glycoprotein precursor protein was explored in detail, and a signal peptide and cleavage were detected at the NH2 terminal region, three transmembrane sites, three putative glycosylation sites, and a highly conserved Gn/Gc cleavage region at the 217 aa position (Additional file 3: Figure S11.E), suggesting that Gn, as expected, is reduced in length. No evidence of NSm protein was found in RNA2. RNA 3 presents three ORFs, sharing the segment architecture with RNA 3 of WMV1. Only the ORF2 351 aa product shared sequence similarity with the nucleoprotein of WMV1 (52% aa identity; E-value = 3e-107, query coverage = 98%). ORF1 encodes a 123 aa protein with similarity with an unannotated product of WMV1, which has been suggested to be a putative NSs [82]. Lastly, ORF3 encodes a 106 aa product with no similarity with other virus (or insect) proteins. The 3′ termini of RNA1–3 shared a 100% conserved stretch of 14 nt, which in *Bunyavirales* is associated to replication [83]. This exact terminus was observed also in RNA1 of WMV1 (Additional file 3: Figure S11.D). Given the intrinsic structural and functional features detected in the virus sequences, we tentatively propose that they correspond to a new virus, which we dubbed *A. triannulatus orthophasmavirus* (AtOPV). In order to entertain this hypothesis, we generated phylogenetic insights of the putative virus using the predicted RdRP of AtOPV and recognized/proposed members of the *Bunyavirales* order in multiple alignments and maximum likelihood trees (Additional file 3: Figure S11.F-H). Unequivocally, AtOPV clustered within the *Phasmaviridae* family (Additional file 3: Figure S11.F). Local topology of the obtained tree suggests that AtOPV clades among a distinctive sub-group of viruses hosted in both mosquitoes (*Culex tritaeniorhynchus*) and midges (*Chaoborus trivitattus*), including KPOPV, WMV1 and WMV2 (Additional file 3: Figure S11.G-H). Our results expand the distribution of phasmaviruses to South America, and suggest that Anophelines are also host of these insect-specific viruses. Future studies should focus on assessing the prevalence and potential effect of virus presence in *A. triannulatus*.

### *Novel anphevirus are hosted by* A. marajoara *and A. darlingi*

The recently recognized *Anphevirus* genus corresponds to an unassigned family within the *Mononegavirales* order. The last International Committee on Taxonomy of Viruses (ICTV) report [84] indicates there is only one member species: *Xincheng anphevirus* including the Xincheng mosquito virus (XcMV) associated to *A. sinensis*, which was described in a large metagenomics study focusing in ssRNA(−) invertebrate viruses [82]. The *Anphevirus* genus appears to be closely related to members of *Bornaviridae* and *Nyamiviridae* family. A number of novel Anphevirus-like viruses have been described recently in West Australian *Culex* mosquitoes [85], West African *A. gambiae* mosquitoes [86], and *Aedes aegypti* mosquitoes [87]. Overall, anpheviruses have been characterized by monosegmented 11–12 kb long negative-stranded RNA genomes encoding in its antigenome six/seven non-overlapping ORFs associated with nucleoproteins (N), small transmembrane proteins (STM), glycoproteins (G1–2), small ZnF proteins (ZnF), and large RdRPs. *Anphevirus,* like other *Mononegavirales*, have their ssRNA(−) genomes encapsidated within the nuclecapsid and the RNA polymerase complex. Their RNA genome is used by the RdRP as a template to transcribe discrete mRNAs from the subgenomic genes, which are separated by gene junctions. These mRNAs are capped and polyadenylated. In our bulk BLASTX searches against the NCBI refseq viral protein database, two contigs from *A. marajoara* (12,764 nt) and *A. darlingi* (13,063 nt) showed significant hits with the RdRP of Bolahun virus (BV, 41–46% identity, E-value = 0). BV is a recently proposed anphevirus found in *A. gambiae* mosquitoes from Burkina Faso (variant 1) and Liberia (variant 2), sharing a 94.2% nt pairwise identity [86].

After contig curation by read mapping, two viral like sequences emerged, harboring the genomic organization of anpheviruses, which we cautiously designated as *A. marajoara* virus (AnMV, 12,265 nt) and *A. darlingi* virus (AnDV, 12,521 nt). The overall pairwise nucleotide identity between AnMV and AnDV was 61%. Sequence annotation revealed that both sequences presented in their putative antigenomes six ORFs encoding typical anpheviruses predicted gene products in their canonical architecture: 3′-N-STM-G1-G2-ZnF-RdRP-5′ (Additional file 3: Figure S12.A). ORF1 of AnMV and AnDV encode a 424–428 aa protein sharing 49.2% identity among them and a 30–29% similarity with their closest hit: Aedes anphevirus (AeAV) and Gambiae virus N proteins, respectively. HHPred searches suggested that both AnMV and AnDV Ns shared protein homology with the p40 nucleoprotein of the Borna disease virus (BoDV-NP, E-value = 3.4e-5, probability 98.3%), a feature which was also associated to the AeAV N [87]. ORF2 AnMV and AnDV products are short 120–121 aa with two or one clear transmembrane domains, respectively, sharing a 29% pairwise aa similarity (the most divergent gene product among both viruses). The putatively 426–457 aa glycoproteins encoded in ORF3 of AnMV and AnDV had no significant hits in both HHPred or BLASTP searches and shared 29.8% pairwise aa identity among them. ORF4 of AnMV and AnDV encode 647–639 aa glycoproteins, presenting signal peptides in their N-terminus, followed by O- and N-linked glycosylated sites and two transmembrane domains in the C-terminus of the protein. AnDV presents an additional transmembrane domain in the N-region. Both glycoproteins shared a 71.8% aa pairwise similarity and 50–51% aa similarity to the G2 of Bolahun virus and Gambiae virus. In addition, as for the G2 of AeAV [87], both AnMV and AnDV G2 reported significant protein homology with the Human Herpesvirus 1 Envelope Glycoprotein B (HHV1-gB, E-value = 1e-10 and 1e-17, probability 98.1–97.5%). ORF5 encode a short 46–48 aa product with zinc ribbon type zinc finger domains which were evident when aligned with other anphevirus equilocal products by the significant conservation of Cysteine CXXC motifs (Additional file 3: Figure S12.H). Lastly, ORF6 encodes a ca. 230 kDa RdRP, 2013–2018 aa long, presenting the expected *Mononegavirales* RdRP (Mononeg_RNA_Pol, E-value = 1.06e-63), mRNA capping (M_ne_mRNAcap, E-value = 2.54e-11) and guanine-7-methyltransferase (SAM_MT_MNV_L, E-value = 1.92e-10) domains. AnMV and AnDV RdRPs share a 66.9% aa pairwise identity and 50–51% similarity with other anpheviruses.

Interestingly, while exploring the intergenic regions of AnMV and AnDV and other anphevirus we were able to pinpoint and characterize a conserved pattern of gene junctions separating the six/seven gene products. A central nucleotide conserved sequence was identified: reading in the antigenome as 3′-UAAAAAACCCGCUAGUUA-5′ tentatively functioning as polyadenylation signal terminating each cistron (P(A)Sig), spacer sequence (Sp) and transcription start site (Tstart) of the next mRNA species (Additional file 3: Figure S12.F). With minor variations, this oligomer was found in most gene junctions of all anpheviruses reported yet (Additional file 3: Figure S12 G).

While polishing the AnDV sequence using our datasets we became aware that the virus was present in the RNA libraries corresponding to both the *A. darlingi* mosquitoes’ samples collected from Macapá (State of Amapá), and Manaus (State of Amazonas) (Additional file 3: Figure S12.I). Moreover, given the low intra-variability of viral reads in each sample (below 0.8%) we were able to assemble and easily differentiate the RNA virus genomes associated with the mosquitoes of both locations with high confidence (mean coverage of 2195/5371X; total virus reads 219,718/538083; 1488/4604 RPKM for AnDV-Mac and AnDV-Man, respectively), which shared an overall 94.7% sequence identity at the nucleotide level. Additionally, we observed that most polymorphism corresponded to silent SNPs, concomitant with higher sequence similarity for the predicted products at the aa level and accompanied with high sequence conservation at both the 3′ leader regions and the conserved gene junctions (Additional file 3: Figure S12 B).

Overall, these observations suggest that the observed variability is biological, and not artifactual. It is interesting that we observed such an elevated level of variation among strains of geographically distinct isolates. It is tempting to suggest that this could be evidence that the *A. darlingi* mosquito populations of both localities have been separated for an extended time limiting the horizontal transmission and eventual homogenization of virus populations. Future studies should provide insights into the phylogenomics aspects of these viruses.

To assess the evolutionary landscape of the described viruses, we generated phylogenetic insights of the putative anpheviruses using their predicted RdRPs in multiple alignments and maximum likelihood trees with replicase proteins of virus members of the *Mononegavirales* order (Additional file 3: Figure S12.C). Both AnMV and AnDV RdRPs clustered in a sub-phylloclade of anpheviruses and other unclassified invertebrate viruses, having as divergent sister clades bornaviruses and nyamiviruses, as expected. Local topology of the obtained phylogenetic tree suggests that AnMV and AnDV are closely related and cluster among a sub-clade within anphevirus conformed also by BV and Gambiae virus (Additional file 3: Figure S12.D-E). The branching of AnMV and AnDV within a clade circumscribed to mosquito-specific viruses provides further support of the nature of the detected RNA sequences as evidence of bona fide mosquito viruses. Overall, our results provide evidence of novel anpheviruses associated to the Amazonian anophelines *A. marajoara* and *A. darlingi*.

## Conclusions

Transcriptomic, proteomic, and genomic studies have considerably improved, in the last ten to fifteen years, our understanding of complexity and functions of blood feeding insect salivary proteins. As far as the family Culicidae is concerned, 12 sialotranscriptomes are currently available, six of which on anopheline mosquitoes: four from Old world (*A. gambiae*, *A. coluzzii*, *A. funestus,* and *A. stephensi*) and two from New world species (*A. albimanus* and *A. darlingi*) [88]. Despite this considerable progress there is still much to learn since we still completely ignore the function of almost 40% of the putative mosquito salivary proteins identified thus far. Besides their pharmacological properties, which may be exploited for the development of new drugs (e.g. anti-thrombotics), salivary proteins of blood feeding arthropods may be turned into tools to prevent and/or better control vector borne diseases, for example, through the development of vaccines or biomarkers to evaluate human exposure to vector bites [89–91]. The sialotranscriptome study reported here provided novel data on four New World anopheline species and allowed us to extend our knowledge on the salivary repertoire of *A. darlingi*. This information may be helpful not only for the characterization of novel activities, but also for the identification of salivary biomarkers to evaluate human exposure to malaria vectors in Central and South America. This may be especially relevant since the *A. gambiae* gSG6, which proved to be a reliable marker of exposure to African [92–94], Asian [95], and Melanesian [96] malaria vectors it is not found in New World anopheline species, as suggested earlier [30] and confirmed by the present study. Additionally, we discovered novel viruses following analysis of the transcriptomes, a procedure that should become standard within future RNAseq studies.

## Methods

### Mosquitoes

The five anopheline species used in this study were collected in the Brazilian Amazon region (Fig. 1). The samples of *A. darlingi* were collected in two distinct localities: 1) in the District of Coração (N 0° 01′; W 51° 10′), located at km 13 of the Duca Serra Road, in the outskirts of the Macapá city, state of Amapá; 2) in the Ramal do Brasileirinho (S 3° 20′ 08.7″; W 59° 52′ 16.8″) located on the outskirts of city of Manaus, state of Amazonas. These localities are separated ~ 1100 km apart and in both localities there is malaria transmission. Samples of *A. braziliensis* were collected from the outskirts of Macapá city (N 0° 1′ 13.71″; W 51° 09′ 47.47″), whereas *A. marajoara and A. triannulatus* were collected in the Santa Bárbara Farm (N 0° 17′ 28.4″; W 50° 54′ 07.2″), municipality of Macapá, state of Amapá, Brazil. Specimens of *A. nuneztovari* were captured in the Ramal do Sampaio (S 03° 41′ 51.8″; W 59° 07′ 37.5″), municipality of Autazes, state of Amazonas, Brazil. The salivary glands of *A. darlingi*, *A. braziliensis*, *A. marajoara,* and *A. triannulatus* used in this study were collected from wild mosquitoes (females) captured from the field, whereas those from *A. nuneztovari* were female descendants from the F_1_ generation reared in the insectary from wild mosquitoes (females) collected from field. The collections were authorized by the System of Authorization and Information in Biodiversity (SISBIO), with permanent license number 38440–1 awarded to VMS.

Adult mosquitoes were captured using a light trap, white Shannon-type, between 18:00 and 22:00 h, and then transferred into cups, properly labeled with locality and collection date. At the end of the captures, the cups containing the mosquitoes were covered with moistened towel paper and transported alive inside tightly closed isothermal boxes to the Laboratório de Genética de Populações e Evolução de Mosquitos Vetores at the Instituto Nacional de Pesquisas da Amazônia (INPA), in Manaus, Brazil. The following day, the specimens were identified using morphological keys of Forattini [15] and Faran and Linthicum [97]. Immediately after, the mosquitoes were cooled in a freezer at − 20 °C for a few minutes, transferred to an ice-chilled plate, when their salivary glands were dissected on a slide containing a drop of sterile Phosphate Buffered Saline (PBS) pH 7.4, under a stereomicroscope, SV11 model, Carl Zeiss. The salivary glands of each species were immediately transferred to an Eppendorf tube containing 200 μL of RNAlater (Thermo Fisher Scientific) solution. Depending on the species analyzed, pools ranging from 80 to 97 pairs of salivary glands were dissected, kept at 4^o^ C for 48 h and then stored at − 80 °C until the RNA extraction. For more details, see Additional file 1: Table S1.

### RNA preparation

Total RNA from salivary glands was extracted using an RNeasy mini total RNA isolation kit (Qiagen, USA), according to the manufacturer’s protocol.

### cDNA library construction and sequencing

Tissue samples were submitted to the North Carolina State Genomic Sciences Laboratory (Raleigh, NC, USA) for Illumina RNA library construction and sequencing. Prior to library construction, RNA integrity, purity, and concentration were assessed using an Agilent 2100 Bioanalyzer with an RNA 6000 Nano Chip (Agilent Technologies, USA). Purification of messenger RNA (mRNA) was performed using the oligo-dT beads provided in the NEBNExt Poly(A) mRNA Magnetic Isolation Module (New England Biolabs, USA). Complementary DNA (cDNA) libraries for Illumina sequencing were constructed using the NEBNext Ultra Directional RNA Library Prep Kit (NEB) and NEBNext Mulitplex Oligos for Illumina (NEB) using the manufacturer-specified protocol. Briefly, the mRNA was chemically fragmented and primed with random oligos for first strand cDNA synthesis. Second strand cDNA synthesis was then carried out with dUTPs to preserve strand orientation information. The double-stranded cDNA was then purified, end repaired, and “a-tailed” for adaptor ligation. Following ligation, the samples were selected a final library size (adapters included) of 400–550 bp using sequential AMPure XP bead isolation (Beckman Coulter, USA). Library enrichment was performed and specific indexes for each sample were added during the protocol-specified PCR amplification. The amplified library fragments were purified and checked for quality and final concentration using an Agilent 2100 Bioanalyzer with a High Sensitivity DNA chip (Agilent Technologies, USA). The final quantified libraries were sequenced in an Illumina HiSeq 2500 DNA sequencer, utilizing 125 bp single end sequencing flow cell with a HiSeq Reagent Kit v4 (Illumina, USA). One lane was run for both *A. darlingi* libraries, and another lane was run with the remaining four libraries. Flow cell cluster generation for the HiSeq2500 was performed using an automated cBot system (Illumina, USA). The software package Real Time Analysis (RTA), version 1.18.64, was used to generate raw bcl, or base call files, which were then de-multiplexed by sample into fastq files for data submission using bcl2fastq2 v2.16.0 software.

### Bioinformatic analysis

Bioinformatic analyses were conducted following the methods described previously [76, 78], with some modifications. Briefly, the fastq files were trimmed of low quality reads (< 20), removed from contaminating primer sequences and concatenated for single-ended assembly using the Abyss (using k parameters from 21 to 91 in 5 fold increments) [98] and Trinity [99] assemblers. These two assemblers were used, because in our previous experience the Abyss assembler produces more novel contigs than the Trinity assembler, but unfortunately it tends to misassembly abundant contigs apparently due to inclusion of products containing introns. However, these abundantly expressed contigs are properly assembled by Trinity. The combined fasta files were further assembled using a iterative blast and CAP3 pipeline as previously described [100]. In the case of *A. darlingi*, the deducted coding sequences (CDS) of the assembled genome (downloaded from Vector Base) [101] were added to the last assembly stage. CDS were extracted based on the existence of a signal peptide in the longer open reading frame (ORF) and by similarities to other proteins found in the Refseq invertebrate database from the National Center for Biotechnology Information (NCBI), proteins from Diptera deposited at NCBI’s Genbank and from SwissProt. Accordingly, from ~ 6 to 38 thousand CDS were extracted from each of the five assemblies, varying in average from 615 to 1200 nt in length (Additional file 1: Table S4). The *A. nuneztovari* library produced the poorest assembly due to ~ 25% rRNA contamination. The reads mapping to rRNA were excluded from the analysis.

Reads for each library were mapped on the deducted CDS using blastn with a word size of 25, 1 gap allowed and 95% identity or better required. Up to five matches were allowed, if and only if, the scores were the same as the largest score. To compare transcript relative expression among contigs, we used the “expression index” (EI) defined as the number of reads mapped to a particular CDS multiplied by 100 and divided by the largest found number of reads mapped to a single CDS. Detection of single nucleotide polymorphisms (SNP’s) were performed with the program Samtools [102] the output of which was used by a program written in visual basic by JMR to assign the synonymous or non-synonymous status of the SNP. The final results were mapped into an excel spreadsheet. Functional classification of the transcripts was achieved by scanning the output of the different blast and rpsblast results using a vocabulary of ~ 400 words, the e value of the result and a result coverage > 75%. The classification of “unknown” was given if no match could be found.

Protein alignments were done using clustalX [103], and phylogenies were inferred using the Mega v.6 package [104], The evolutionary history was inferred by using the Maximum Likelihood method based on the best nucleotide substitution matrix available for the alignment, as discovered by the Mega package. The bootstrap consensus tree inferred from 500 replicates is taken to represent the evolutionary history of the taxa analyzed [105]. Other parameters are as indicated in the figure legends.

### Virus discovery and analyses

Virus discovery and annotation was implemented as described in [106]. In brief, de novo assemblies were subjected to BLASTX searches (E-value = 1e-5) against refseq viral proteins available at ftp://ftp.ncbi.nlm.nih.gov/refseq/release/viral/viral.1.protein.faa.gz. Hits were explored by hand and curated by iterative mapping of reads. ORFs were predicted by ORFinder as implemented in https://www.ncbi.nlm.nih.gov/orffinder/. Functional and structural domains of the predicted gene products were assessed with the NCBI CDD tool https://www.ncbi.nlm.nih.gov/Structure/cdd/wrpsb.cgi and the HHPred tool https://toolkit.tuebingen.mpg.de/#/tools/hhpred. SignalP 4.1 http://www.cbs.dtu.dk/services/SignalP/ was used to detect signal peptides and cleavage regions, TMHMM 2.0 http://www.cbs.dtu.dk/services/TMHMM-2.0/ for transmembrane predictions, and NetNGlyc 1.0 http://www.cbs.dtu.dk/services/NetNGlyc/ for glycosylation site prediction. Abundance of virus reads was calculated by mapping with standard parameters using Bowtie2 http://bowtie-bio.sourceforge.net/bowtie2/index.shtml. Phylogenetic insights were based on multiple alignments of replicase proteins with MAFFT v7 https://mafft.cbrc.jp/alignment/software/ with an E-INS-i iterative refinement method and BLOSUM64 scoring matrix for amino acids. Phylogenetically uninformative sites were trimmed using the GBlocks tool v.0.91b available as a web server at http://molevol.cmima.csic.es/castresana/Gblocks_server.html. Maximum likelihood trees were generated with FastTree v2.1 http://www.microbesonline.org/fasttree/ with JTT models of amino acid evolution, 1000 tree re-samples and local support values estimated with the Shimodaira-Hasegawa test.

## Additional files

Additional file 1: **Tables S1–S7.** in a single file. (DOCX 35 kb)

Additional file 2: Supplemental spreadsheet. (DOCX 11 kb)

Additional file 3: **Figures S1–S12.** in a single web format file. (MHT 7748 kb)

## Abbreviations

AeAV: Aedes anphevirus
AnDV: *A. darlingi* virus
AnMV: A. marajoara virus
AtOPV: *A. triannulatus* orthophasmavirus
BoDV: Borna disease virus
G1-2: Glycoproteins
HHV1: Human Herpesvirus 1
ICTV: International Committee on Taxonomy of Viruses
KPOPV: Kigluaik phantom orthophasmavirus
NOMV: Nome phantom orthophasmavirus
NP: Nucleoprotein
NW: New World
OW: Old World
RdRP: RNA-dependent RNA polymerase
SISBIO: System of authorization and information in biodiversity
STM: Small transmembrane proteins
WMV: Wuhan mosquito virus
XcMV: Xincheng mosquito virus
ZnF: Small ZnF proteins
