# Supplementary material for: An insight into the sialotranscriptome and virome of Amazonian anophelines
Source: BMC Genomics. 2019 Mar 4;20:166. doi: 10.1186/s12864-019-5545-0 (PMC6399984; doi:10.1186/s12864-019-5545-0)
Supplement: Supplementary file 1 — Tables S1–S7. in a single file. (DOCX 35 kb) [file 12864_2019_5545_MOESM1_ESM.docx]

**Supplemental table 1: Species used in this study, their origin and number of salivary glands dissected**

(1) *An. darlingi* collected in Casa do Peri, District of Coração, Municipality of Macapá, Amapá State (81 pairs)

(2) *An. darlingi* collected in Sitio Ryfran, Ramal do Brasileirinho, outskirts of Manaus, Amazonas State (97 pairs)

(3) *An. braziliensis* collected in Trilho do Trem, outskirts of Macapá, Amapá State (80 pairs)

(4) *An. marajoara* collected in Santa Bárbara Farm, Municipality of Macapá, Amapá State (80 pairs)

(5) *An. nuneztovari* collected in Ramal do Sampaio, Autazes, Amazonas State (84 pairs from F1 reared in the lab)

(6) *An. triannulatus* collected in Santa Bárbara Farm, Municipality of Macapá, Amapá State (80 pairs)

**Supplemental table 2: NCBI accessions for Bioproject ID: PRJNA428765**

| **Species** | **Biosample** | **Reads** | **TSA** | **Number of submitted CDS** |
| --- | --- | --- | --- | --- |
| *Anopheles nuneztovari* | [SAMN08332483](https://www.ncbi.nlm.nih.gov/biosample/?term=SAMN08332483) | [SRR6471062](https://www.ncbi.nlm.nih.gov/sra/?term=SRR6471062) | GGFF00000000 | 284 |
| *Anopheles darlingi* from Ryfran | [SAMN08332484](https://www.ncbi.nlm.nih.gov/biosample/?term=SAMN08332484) | [SRR6471061](https://www.ncbi.nlm.nih.gov/sra/?term=SRR6471061) |  |  |
| *Anopheles darlingi* from Peri | [SAMN08332485](https://www.ncbi.nlm.nih.gov/biosample/?term=SAMN08332485) | [SRR6471060](https://www.ncbi.nlm.nih.gov/sra/?term=SRR6471060) | GGFL00000000 * | 16,305 |
| *Anopheles braziliensis* | [SAMN08332486](https://www.ncbi.nlm.nih.gov/biosample/?term=SAMN08332486) | [SRR6471059](https://www.ncbi.nlm.nih.gov/sra/?term=SRR6471059) | GGFM00000000 | 12,570 |
| *Anopheles marajoara* | [SAMN08332487](https://www.ncbi.nlm.nih.gov/biosample/?term=SAMN08332487) | [SRR6471058](https://www.ncbi.nlm.nih.gov/sra/?term=SRR6471058) | GGFJ00000000 | 15,037 |
| *Anopheles triannulatus* | [SAMN08332488](https://www.ncbi.nlm.nih.gov/biosample/?term=SAMN08332488) | [SRR6471057](https://www.ncbi.nlm.nih.gov/sra/?term=SRR6471057) | GGFK01000001 | 15,820 |

***** Submitted a single file for both *An. darlingi* libraries

**Supplemental table 3: Read statistics following primer removal and tips with quality smaller than 20**

| **Library name** | **Total number of sequences** | **Total number of residues** | **Average length** | **Median size** | **L50** | **Smaller than 100 bp** | **Smaller sequence size** |
| --- | --- | --- | --- | --- | --- | --- | --- |
| Anda-peri (1) | 147,564,265 | 18,274,737,685 | 123.8426 | 125 | 125 | 2,694,824 | 30 |
| Anda-Ryfran (2) | 116,857,065 | 14,369,098,588 | 122.963 | 125 | 125 | 3,775,854 | 30 |
| Anbrazz (3) | 41,294,165 | 5,066,753,687 | 122.699 | 125 | 125 | 1,460,989 | 30 |
| Anmarajo (4) | 57,729,051 | 7,153,544,601 | 123.9159 | 125 | 125 | 975,348 | 30 |
| Annunez (5) | 76,883,466 | 9,527,129,473 | 123.9165 | 125 | 125 | 1,306,633 | 30 |
| Antrian (6) | 46,603,769 | 5,790,007,317 | 124.239 | 125 | 125 | 536,481 | 30 |

(1) *An. darlingi* from District of Coração, State of Amapá

(2) *An. darlingi* from Ramal do Brasileirinho, State of Amazonas

(3) *An. braziliensis*

(4) *An. marajoara*

(5) *An. nuneztovari*

(6) *An. triannulatus*

**Supplemental table 4: Number and average length of coding sequences extracted for each organism**

| **Library** | **Number of coding sequences larger than 200 nt** | **Average length** |
| --- | --- | --- |
| *An. darlingi* | 38,308 | 1,136 |
| *An. braziliensis* | 17,656 | 893 |
| *An. marajoara* | 24,019 | 1,076 |
| *An. nuneztovari* | 6,325 | 615 |
| *An. triannulatus* | 20,885 | 1,201 |

**Supplemental table 5:** Selected putative salivary proteins coded by the sialotranscriptome of *An. darlingi, An. marajorara, An. triannulatus, An. nuneztovari* and *An.braziliensis,* including their average expression index (EI).

| **Class** | **Average EI** | **SE** | **N** | **Function? (1)** | **References** |
| --- | --- | --- | --- | --- | --- |
| **I – Ubiquitous protein families existing outside Nematocera** | |  |  |  |  |
| Enzymes |  |  |  |  |  |
| 5'nucleotidase/Apyrase family | 22.53 | 3.60 | 19 | Y |  |
| Glycosidases | 21.41 | 5.07 | 15 | Y |  |
| Anopheline peroxidases | 16.70 | 3.85 | 16 | Y |  |
| Immunity-related proteins |  |  |  |  |  |
| Gambicin | 2.41 | 1.03 | 4 | Y |  |
| Gram negative binding protein | 2.08 | 1.36 | 2 | Y |  |
| Lysozyme | 1.38 | 0.26 | 7 | Y |  |
| Defensin | 0.02 | 0.01 | 3 | Y |  |
| Mucins |  |  |  |  |  |
| SG3 mucin family | 20.06 | 3.30 | 60 |  |  |
| gSG5 mucin protein family | 4.73 | 1.03 | 11 |  |  |
| Mucin I mosquito family | 2.81 | 0.41 | 21 |  |  |
| Virus induced mucin | 2.72 | 1.14 | 6 |  |  |
| OBP superfamily |  |  |  |  |  |
| Anopheline short D7 family | 26.16 | 3.59 | 57 | Y |  |
| Long D7 mosquito family | 17.70 | 1.76 | 15 | Y |  |
| Antigen-5 gVAg family | 53.35 | 8.66 | 15 |  |  |
| **II - Protein families exclusive of blood sucking Nematocera** | |  |  |  |  |
| 30 kDa/Aegyptin family | 54.97 | 5.02 | 16 | Y |  |
| Canonical 41 kDa family | 8.56 | 1.71 | 12 |  |  |
| Mucin II mosquito family | 1.93 | 0.38 | 6 |  |  |
| **III - Protein families specific of mosquitoes** |  |  |  |  |  |
| cE5/Anophelin family | 24.10 | 4.96 | 15 | Y |  |
| Anopheline SG1 family | 18.50 | 1.59 | 81 |  |  |
| hyp15-17 family | 15.96 | 4.04 | 24 |  |  |
| 56 kDa mosquito family | 7.62 | 2.58 | 6 |  |  |
| Hyp8.2 family | 6.57 | 0.57 | 45 |  |  |
| HHH peptide family | 4.83 | 0.55 | 19 |  |  |
| GGGG family similar to HHH family | 2.48 | 0.30 | 25 |  |  |
| Basic tail mosquito family | 2.84 | [14, 15]0.67 | 13 |  |  |
| 23.5 kDa culicine family | 2.62 | 0.99 | 4 |  |  |
| gSG7 family/Anophensins | 2.12 | 0.21 | 19 | Y |  |
| gSG8 family | 1.98 | 0.45 | 12 |  |  |
| Hyp6.2 family | 1.38 | 0.13 | 18 |  |  |
| hyp8.2 culicine family | 0.62 |  | 1 |  |  |
| Other salivary polypeptides | 0.97 | 0.47 | 26 |  |  |
| **Total** |  |  | **593** |  |  |

1. Indicates whether at least one publication characterizes a function for the protein product.

**Supplemental table 6:** Transcripts coding for members of the Apyrase/5’-nucleotidase family, and their expression indices (EI).

| **Transcript name** | **EI** | **Species** |
| --- | --- | --- |
| [AnbraSigP-146759_FR4_1-582](file:///E:\vera\all\links\pep\AnbraSigP-146759_FR4_1-582-pep.txt) | 48.20 | *An. braziliensis* |
| [Anbra-147290_FR2_1-576](file:///E:\vera\all\links\pep\Anbra-147290_FR2_1-576-pep.txt) | 45.60 | *An. braziliensis* |
| [Anmarj-153169_FR3_1-492](file:///E:\vera\all\links\pep\Anmarj-153169_FR3_1-492-pep.txt) | 38.86 | *An. marajoara* |
| [Anbra-116766_FR3_1-392](file:///E:\vera\all\links\pep\Anbra-116766_FR3_1-392-pep.txt) | 37.71 | *An. braziliensis* |
| [AnnunSigp-39092_FR4_1-583](file:///E:\vera\all\links\pep\AnnunSigp-39092_FR4_1-583-pep.txt) | 35.06 | *An. nuneztovari* |
| [AnnunSigp-38604_FR2_1-577](file:///E:\vera\all\links\pep\AnnunSigp-38604_FR2_1-577-pep.txt) | 31.13 | *An. nuneztovari* |
| [Antri-133769_FR4_1-572](file:///E:\vera\all\links\pep\Antri-133769_FR4_1-572-pep.txt) | 30.65 | *An. triannulatus* |
| [Antri-134380_FR6_1-571](file:///E:\vera\all\links\pep\Antri-134380_FR6_1-571-pep.txt) | 30.56 | *An. triannulatus* |
| [AndaSigp-111604_FR1_24-598](file:///E:\vera\all\links\pep\AndaSigp-111604_FR1_24-598-pep.txt) | 28.95 | *An. darlingi* |
| [AndaSigp-111602_FR4_5-579](file:///E:\vera\all\links\pep\AndaSigp-111602_FR4_5-579-pep.txt) | 27.85 | *An. darlingi* |
| [Andar-111603_FR2_22-619](file:///E:\vera\all\links\pep\Andar-111603_FR2_22-619-pep.txt) | 27.32 | *An. darlingi* |
| [Anmarj-167613_FR5_583-1123](file:///E:\vera\all\links\pep\Anmarj-167613_FR5_583-1123-pep.txt) | 10.83 | *An. marajoara* |
| [AnnunSigp-38029_FR2_1-561](file:///E:\vera\all\links\pep\AnnunSigp-38029_FR2_1-561-pep.txt) | 6.99 | *An. nuneztovari* |
| [Antri-135781_FR6_480-1054](file:///E:\vera\all\links\pep\Antri-135781_FR6_480-1054-pep.txt) | 6.50 | *An. triannulatus* |
| [AntriSigP-135781_FR6_480-1054](file:///E:\vera\all\links\pep\AntriSigP-135781_FR6_480-1054-pep.txt) | 6.50 | *An. triannulatus* |
| [AnnunSigp-37852_FR5_46-625](file:///E:\vera\all\links\pep\AnnunSigp-37852_FR5_46-625-pep.txt) | 5.95 | *An. nuneztovari* |
| [Anbra-145239_FR6_1-564](file:///E:\vera\all\links\pep\Anbra-145239_FR6_1-564-pep.txt) | 5.11 | *An. braziliensis* |
| [Andar-97451_FR6_53-586](file:///E:\vera\all\links\pep\Andar-97451_FR6_53-586-pep.txt) | 2.43 | *An. darlingi* |
| [Anbra-146985_FR3_1-345](file:///E:\vera\all\links\pep\Anbra-146985_FR3_1-345-pep.txt) | 1.88 | *An. braziliensis* |

**Supplemental table 7:** Ratio of non-synonymous to synonymous mutations observed on transcripts with coverage depth higher than 100 and according to their functional class.

|  | *An. darlingi* | *An. triannulatus* | *An. marajoara* | *An. braziliensis* |  |
| --- | --- | --- | --- | --- | --- |
| **Class** | **NS/S** | **NS/S** | **NS/S** | **NS/S** | **Average** |
| Unknown | 1.81 | 1.82 | 1.85 | 1.74 | 1.80 |
| Secreted | 1.88 | 1.74 | 1.88 | 0.61 | 1.53 |
| Transposable element | 0.46 | 0.23 | 1.04 | 0.08 | 0.45 |
| Extracellular matrix | 0.45 | 0.14 | 0.29 | 0.12 | 0.25 |
| Nuclear regulation | 0.26 | 0.18 | 0.19 | 0.30 | 0.23 |
| Detoxification | 0.29 | 0.06 | 0.29 | 0.24 | 0.22 |
| Signal transduction | 0.21 | 0.15 | 0.20 | 0.18 | 0.19 |
| Proteasome machinery | 0.08 | 0.25 | 0.07 | 0.20 | 0.15 |
| Immunity | 0.30 | 0.06 | 0.18 | 0.00 | 0.14 |
| Transcription factor | 0.24 | 0.02 | 0.17 | 0.09 | 0.13 |
| Protein modification machinery | 0.15 | 0.10 | 0.11 | 0.11 | 0.12 |
| Transcription machinery | 0.12 | 0.08 | 0.13 | 0.10 | 0.11 |
| Cytoskeletal | 0.14 | 0.15 | 0.09 | 0.04 | 0.11 |
| Transporters/storage | 0.19 | 0.08 | 0.09 | 0.06 | 0.10 |
| Metabolism | 0.15 | 0.08 | 0.10 | 0.08 | 0.10 |
| Unknown, conserved | 0.18 | 0.06 | 0.09 | 0.05 | 0.10 |
| Protein export machinery | 0.04 | 0.04 | 0.14 | 0.05 | 0.07 |
| Protein synthesis machinery | 0.05 | 0.02 | 0.07 | 0.03 | 0.04 |
