# Supplementary material for: An insight into the sialotranscriptome and virome of Amazonian anophelines
Source: BMC Genomics. 2019 Mar 4;20:166. doi: 10.1186/s12864-019-5545-0 (PMC6399984; doi:10.1186/s12864-019-5545-0)
Supplement: Supplementary file 2 — Supplemental spreadsheet. (DOCX 11 kb) [file 12864_2019_5545_MOESM2_ESM.docx]

Link to supplemental spreadsheet S1:

https://s3.amazonaws.com/proj-bip-prod-publicread/transcriptome/Amazonian_anophelines/S1-spreadsheet.zip
